# Recent global trends in the prevalence and incidence of dementia, and survival with dementia

**DOI:** 10.1186/s13195-016-0188-8

**Published:** 2016-07-30

**Authors:** Martin Prince, Gemma-Claire Ali, Maëlenn Guerchet, A. Matthew Prina, Emiliano Albanese, Yu-Tzu Wu

**Affiliations:** 1 The Global Observatory for Ageing and Dementia Care, Health Service & Population Research Department, Institute of Psychiatry, Psychology and Neuroscience, King’s College London, PO 36, David Goldberg Centre, De Crespigny Park, London, SE5 8AF UK; 2 Department of Public Health and Primary Care, School of Clinical Medicine, University of Cambridge, Cambridge, UK; 3 Department of Psychiatry, University of Geneva, Geneva, Switzerland; 4 Cambridge Institute of Public Health, University of Cambridge, Cambridge, UK

**Keywords:** Dementia, Trends, Epidemiology, Projection, Global health, Worldwide, Systematic review, Meta-analysis

## Abstract

**Background:**

Current projections of the scale of the coming dementia epidemic assume that the age- and sex-specific prevalence of dementia will not vary over time, and that population ageing alone (increasing the number of older people at risk) drives the projected increases. The basis for this assumption is doubtful, and secular trends (that is, gradual decreases or increases in prevalence over long-term periods) are perfectly plausible.

**Methods:**

We carried out a systematic review of studies of trends in prevalence, incidence and mortality for people with dementia, conducted since 1980.

**Results:**

We identified nine studies that had tracked dementia prevalence, eight that had tracked dementia incidence, and four that had tracked mortality among people with dementia. There was some moderately consistent evidence to suggest that the incidence of dementia may be declining in high-income countries. Evidence on trends in the prevalence of dementia were inconsistent across studies and did not suggest any clear overall effect. Declining incidence may be balanced by longer survival with dementia, although mortality trends have been little studied. There is some evidence to suggest increasing prevalence in East Asia, consistent with worsening cardiovascular risk factor profiles, although secular changes in diagnostic criteria may also have contributed.

**Conclusions:**

We found no evidence to suggest that the current assumption of constant age-specific prevalence of dementia over time is ill-founded. However, there remains some uncertainty as to the future scale of the dementia epidemic. Population ageing seems destined to play the greatest role, and prudent policymakers should plan future service provision based upon current prevalence projections. Additional priorities should include investing in brain health promotion and dementia prevention programs, and monitoring the future course of the epidemic to chart the effectiveness of these measures.

## Background

Almost all current projections of the scale of the coming dementia epidemic, including those published by Alzheimer’s Disease International (ADI) [1–3], assume that the age- and sex-specific prevalence of dementia will not vary over time, and that population ageing alone (increasing the number of older people at risk) drives the projected increases [1, 2, 4, 5]. The basis for this assumption is doubtful, and secular trends (that is, gradual decreases or increases in prevalence over long-term periods) are perfectly plausible [6]. The prevalence of any condition (the proportion of the population affected at a point in time) is a product of its incidence and the average duration of the disease episode. The incidence is the rate at which new cases develop within the population. The duration of dementia equates to time from incidence to death, given that recovery is, sadly, not possible. Changes in either or both of these indicators could lead to changes in age-specific prevalence [1].

It should be noted that trends in the two indicators may not move in the same direction; for example, reductions in incidence might be accompanied by increases in duration of survival with dementia, or vice versa, the one effect tending to cancel out the other in terms of their overall impact on prevalence. Secular trends may vary among world regions, and among different population subgroups within one country. Experience with changing rates of cardiovascular disease, obesity, diabetes and cancer shows this clearly. Geographic or year of birth variations in adult disease occurrence can be explained by differential exposure during different epochs in life, since as early as conception. Hence, the considerable variability in secular trends for these chronic diseases reflects different degrees of progress in improving public health and access to healthcare, and in strengthening health systems and services to better detect, treat and control these conditions.

A decline in age-specific incidence of dementia, at least in high-income countries, is theoretically possible, driven by changes in exposure to suspected developmental, lifestyle and cardiovascular risk factors for dementia [6]. The 2014 World Alzheimer Report focused upon dementia risk reduction, examining the evidence base for modifiable risk factors for dementia [7]. The strongest evidence for possible causal associations with dementia was for low education in early life, hypertension in midlife, and smoking and diabetes across the life course. In a recent modelling exercise, it was estimated that a 10 % reduction in these and other key risk exposures would lead to an 8.3 % reduction in the prevalence of dementia through 2050, with a 15.3 % reduction in dementia prevalence anticipated in response to a 20 % reduction in exposure prevalence [8]. In most world regions, each generation is better educated than the one before. Although trends differ between countries, sexes, age groups and time periods, there has been a general trend in many high-income countries towards less smoking, falling total cholesterol and blood pressure levels, and increasing physical activity [9]. On the other hand, the prevalence of obesity and diabetes has been increasing in most developed countries. The picture in many low- and middle-income countries is quite different: The trends in cardiovascular health among older people are in an adverse direction [9], with a pattern of increasing stroke [10] and ischaemic heart disease morbidity and mortality [11–13], linked to an epidemic of obesity and increasing blood pressure levels [14]. After a lag period, to the extent that these factors are genuinely causally associated with dementia, one would expect to see corresponding reductions (or increases) in the incidence of dementia.

Secular trends in survival with dementia are difficult to measure. Because dementia has a long and highly variable latency period, estimates from clinical services are confounded by time of diagnosis. If diagnosis is being made at an earlier stage in the disease process, then duration of dementia may appear to be increasing, whereas this may signify only that people with dementia are in contact with services for a higher proportion of the overall disease duration. Trends in cause of death on death certificates provide information on secular changes in the attribution of dementia as a cause of death, but not on the all-cause mortality rate among people with dementia [15]. A proper understanding of trends in survival with dementia will come only from monitoring all-cause mortality rates of those with and without the disease, and the ratio between them (standardised mortality ratio, or hazard ratio) over time. Mortality rates among older people continue to fall in all world regions, and for all age groups, accounting for impressive gains in life expectancy from age 60 years [16]. This is now one of the main drivers of population ageing, particularly but not exclusively in higher-income countries. Whether these overall trends for declining mortality apply equally to people living with dementia is not known. Mortality rates among older people are much higher for those living with dementia. In the 10/66 Dementia Research Group studies in Latin America, India and China, after controlling for age and sex, in a Cox proportional hazards regression, hazard of death was 1.56 to 5.69 times higher in those with dementia (meta-analysed HR 2.80, 95 % CI 2.48–3.15) [17]. Effect sizes from studies in countries with low or middle incomes (for example, HR 2.83 [95 % CI 1.10–7.27] in Nigeria [18] and HR 5.16 [95 % CI 3.74–7.12] in Brazil [19]) have tended to be slightly higher than those indicated by a meta-analysis of studies principally from countries with high incomes (relative risk 2.63, 95 % CI 2.17–3.21) [20]. If age-standardised mortality rates among people with dementia decline at the same rate as for those without dementia (that is, the adjusted mortality ratio remains constant over time), survival with dementia, and hence disease duration, will increase progressively.

Finally, it should be noted that one of the indications of successful dementia risk reduction may be that the incidence of dementia is deferred to older ages. Thus, the average age of onset may increase over time. Under these circumstances, age-specific or age-standardised mortality for people with dementia may not change, but overall, for all people with dementia, mortality may be higher and survival with dementia shorter, reflecting that onset is occurring closer to the ‘natural’ end of life. Langa has described this phenomenon as ‘the compression of cognitive morbidity’ [21], a desirable outcome for public health and individual quality of life, resulting in longer, healthier lives, with fewer years spent in a state of reduced independence and needing care.

At the time of the 2009 World Alzheimer Report, what very few data were available from certain high-income countries did not suggest any clear pattern of a decline or increase over time in either the incidence or prevalence of dementia [1, 22, 23]. Our meta-analysis of European studies conducted since 1980 also did not suggest any secular trend in prevalence [4]. Just a few years later, and linked to a greatly increased interest in the potential for prevention of dementia by targeting modifiable risk factors [24, 25], the quality and extent of the evidence has expanded greatly, with reports from several studies of trends in dementia prevalence, incidence and/or mortality within defined populations. Such secular comparisons are conceivably more valid because identical or very similar research methodology has been applied within studies over time. Our aim in this paper is to review this evidence, together with regional meta-analyses of trends in dementia prevalence over time. A preliminary version of this review was published in the World Alzheimer Report 2015 [3]. The present version is substantially updated, taking account of studies published since then and other conference abstracts that have now been published as definitive peer-reviewed papers, with different, and, presumably, more appropriate results.

## Methods

Studies of secular trends since 1980 in the prevalence or incidence of dementia or Alzheimer’s disease within defined populations were identified from the systematic review of studies of dementia prevalence and incidence conducted by the Global Observatory for Ageing and Dementia Care for the World Alzheimer Reports of 2009 [1] and 2015 [3], and from the World Health Organization 2012 report [26], as well as by hand-searching the references of those relevant studies identified. Any studies that met the sampling and ascertainment eligibility criteria for the reviews of prevalence and incidence were included in this review, with the additional inclusion criterion that methodologies within studies should have been held constant between successive prevalence or incidence waves. We did not stipulate any minimum or maximum interval between waves. We report the crude percentage reduction or increase in prevalence or incidence, and rate of change per year, together with adjusted rate ratios (or hazard or odds ratios) if provided, with adjustment for compositional factors, principally age and sex. For mortality, we conducted an additional search of the PubMed database using the search terms “(dementia or alzheim*) AND (mortality or survival) AND trend*”.

## Results

### Dementia prevalence

We identified nine studies that had tracked changes in dementia prevalence over time (Table 1). In one study, the Medical Research Council Cognitive function and Ageing Study (MRC CFAS) [27], there was a statistically significant decline in the prevalence of dementia between 1993 and 2011. This was consistent with a somewhat larger but statistically non-significant decline in the prevalence of dementia in Zaragoza, Spain [28], and with a decline in the prevalence of moderate to severe cognitive impairment seen in the Health and Retirement Study (HRS) in the United States [21]. The annual rates of relative change in prevalence were −1.7 %, −3.6 % and −3.2 % per year, respectively. Set against this, three other studies from Sweden [29, 30] and the United States [31] indicated a stable prevalence of dementia, consistent with short-term trends in German insurance claims data [32]. In a third Swedish study of short-term trends in dementia prevalence among the oldest old, prevalence had increased by 40 % between 2001 and 2006 [33]. In the Japanese Hisayama study, there was a non-significant 38 % relative increase in the prevalence of dementia between 1985 and 2005, with a marked increase in the proportion of cases accounted for by Alzheimer’s disease [34]. This is consistent with findings from one other Japanese study of secular trends, with a 23 % increase in the prevalence of dementia between 1980 and 2000 [35]. This study was excluded from this review because its ascertainment procedures did not meet the minimum quality criteria we set for our global estimates of dementia prevalence [1]. However, although inadequate, they were held constant between the three waves of the study.

**Table 1:** Studies estimating changes in prevalence of dementia or Alzheimer’s disease over time

Study, setting, age range	Outcomes	Relative change (%)	Period	Interval (years)	Relative change (%) per year	Other findings/notes
1. United Kingdom, MRC CFAS, 65 years and older [27]	Dementia (GMS-AGECAT)	30 % reduction AOR 0.7 (0.6–0.9)	1993–2011	18 years	−1.7 %	Bigger dementia prevalence reduction in older age groups. Reduction in the proportion of older people, and people with dementia living in care homes. Increased prevalence of dementia among care home residents.
2. Zaragoza, Spain, 65 years and older [28]	Dementia (DSM-IV)	Non–significant 25 % reduction AOR 0.75 (0.56–1.02) Women AOR 1.02 (0.69–1.51) Men AOR 0.40 (0.25–0.65)	1988–1995	7 years	−3.6 %	Bigger (and statistically significant) dementia prevalence reduction in men. No changes observed in education level.
3. HRS, nationally representative, United States, 70 years and older [21]	Moderate/severe cognitive impairment (probable dementia)	29 % reduction AOR 0.65 (0.58–0.73)	1993–2002	9 years	−3.2 %	Increases in levels of education, significantly fewer IADL limitations but higher rates of cardiovascular risk factors and cardiovascular disease, including diabetes, hypertension, obesity and heart disease. Education differences accounted for 43 % of the prevalence difference between time points. Residents of care homes were excluded from the 1993 wave. The 6.2 % of 2002 respondents who were residents of care homes were also excluded from the comparative analysis. This may have biased the comparison, if transition to care homes was reserved for those with more severe dementia at the later time point.
4. United States, Indianapolis, IN, African Americans, 65 years and older [31]	Dementia (DSM-III-R) AD	Stable 6.8 % vs. 7.5 % (dementia *p* = 0.35) 5.5 % vs. 6.8 % (AD *p* = 0.26)	1991–2002	11 years	No trend	Increases in levels of hypertension, diabetes and stroke, but also higher levels of treatment, consistent with national trends for African Americans over this time period. Some differences in recruitment procedures, and a higher refusal rate in 2002.
5. Stockholm, Sweden, 75 years and older [29]	Dementia (DSM-III-R)	Stable 17.5 % vs. 17.9 % AOR 0.85 (0.68–1.05)	1988–2002	14 years	No trend	Much higher levels of education at the second time point.
6. Germany, insurance claims data, 65 and older [32]	Dementia (ICD-10)	Stable prevalence in all age groups and both sexes, other than women aged 75–84 years AOR 0.97 (0.95–0.98)	2007–2009	3 years	−1.2 % (women aged 75–84 years)	This study used claims data of the largest public health insurance company in Germany. The data contained complete inpatient and outpatient diagnoses according to ICD-10. For the analysis of prevalence, age-specific prevalence was estimated for the years 2007, 2008 and 2009. Secular trends in clinical diagnosis or help-seeking cannot be excluded.
7. Goteborg, Sweden, aged 70 and 75 years [30]	Dementia historical criteria	Stable prevalence for both age groups Age 70 years M 1.7 % vs. 0.9 % F 2.2 % vs. 3.7 % Age 75 years M 6.8 % vs. 6.9 % F 3.8 % vs. 5.3 %	Aged 70 years 1976–2000 Aged 75 years 1976–2005	Aged 70 years 25 years Aged 75 years 30 years	No trend	Higher education level, better results on cognitive tests, better socioeconomic status, better treatment of vascular risk factors and better general physical health in the later-born cohorts
8. Umea, Sweden, 85 years and older [33]	Dementia (DSM-IV)	40 % increase (*p* = 0.001)	2001–2006	5 years	+8.0 %	Prevalence differences not adjusted for other covariates, but age distribution was similar. Increase in the prescription of antihypertensive and statin drugs, cholinesterase inhibitors, and more heart surgery.
9. Japan, Hisayama, aged 65 years and older [34]	Dementia, AD	38 % increase (dementia) AOR 1.34 (0.97–1.87) 255 % increase (AD) 3.28 (1.75–6.14)	1985–2005	20 years	+1.9 % (dementia) + 12.8 % (AD)	Ratio of AD/VaD increasing from 0.5 in 1985 to 1.4 in 2005.

*AD* Alzheimer’s disease, *AGECAT* Automated Geriatric Examination for Computer Assisted Taxonomy, *AOR* adjusted odds ratio, DSM *Diagnostic and Statistical Manual of Mental Disorders*, *GMS* Geriatric Mental State Examination, *HRS* Health and Retirement Study, *IADL* instrumental activities of daily living, *ICD* International Classification of Diseases, *MRC CFAS* Medical Research Council Cognitive function and Ageing Study, *VaD* vascular dementia

### Dementia incidence

Nine studies had tracked dementia incidence over time (Table 2). Statistically significant reductions in the incidence of dementia were reported in two U.S. population-based studies: one of African Americans in Indianapolis, IN [36] and the other derived from the Framingham study [37]. Dementia incidence over time was also tracked in one study done in Bordeaux, France [38]. The annual rates of relative change (−5.5 %, −1.6 % and −3.5 %, respectively) are broadly consistent with a non-significant −2.5 % annual rate of relative change in incidence reported in the Rotterdam study [39]. A similar annual rate of decline in dementia incidence (−3.0 %) was reported in an analysis of German insurance claims data [40], but with only a 3-year interval between the midpoints of the two follow-up periods and with a possibility that trends in help-seeking or clinical diagnosis might have explained the findings. A similar study using comprehensive health information system data for the Canadian province of Ontario suggested a −0.6 % decline in standardised incidence over a 12-year period (2002–2013) [41]. To the extent that changes in incidence can be inferred from changes in prevalence and mortality, data from repeated surveys in Stockholm, Sweden, are also consistent with a decline in dementia incidence [29]. On the other hand, population-based studies conducted in Chicago, IL, USA [31], and Ibadan, Nigeria [36], indicated a stable incidence of dementia over 11-year periods. One further study, in which researchers reported a stable incidence of dementia in Beijing, China, was excluded from the review because it used slightly different diagnostic criteria at the two time points [42].

**Table 2:** Studies estimating changes in the incidence of dementia or Alzheimer’s disease over time

Study, setting, age range	Outcomes	Relative change (%)	Period	Interval between incidence cohorts (years)	Relative change (%) per year	Other findings
Directly observed						
1. Indianapolis, IN, USA, African Americans, 65 years and older [36]	Dementia (DSM-III-R) AD	Dementia 3.6 % per annum (3.2–4.1 %) vs. 1.4 % per annum (1.2–1.7 %) 61 % reduction AD 2.5 % per annum (2.1–2.9 %) vs. 1.3 % per annum (1.0–1.5 %) 48 % reduction	1991–2002	11 years	Dementia −5.5 % AD −4.4 %	Biggest reduction in youngest age groups. See also notes for study 4 in Table 1.
2. Framingham, MA, USA, 60 years and older [37]	Dementia DSM-IV AD (NINCDS-ADRDA) VaD (NINDS-AIREN); diagnoses by consensus review panel	Dementia 44 % reduction AHR 0.56 (0.41–0.77) AD 30 % reduction AHR 0.70 (0.48–1.03) VaD 55 % reduction AHR 0.45 (0.23–0.87)	1980–2006	26 years	Dementia −1.7 % AD −1.2 % VaD −2.1 %	Biggest reduction in youngest age groups. No reduction among the least educated. Significant improvements in education status; use of antihypertensive and statin medication; blood pressure and HDL levels; and prevalence of smoking, heart disease and stroke; however, prevalence of obesity and diabetes increased.
3. Bordeaux, France, 65 years and older [38]	*Algorithm diagnosis* (using MMSE score and IADL only) *Clinical diagnosis* ‘based upon’ DSM-IIIR/DSM-V	*Algorithmic diagnosis* Overall AHR 0.65 (0.53–0.81) Women AHR 0.62 (0.48–0.80) Men AHR 1.10 (0.69–1.78) *Clinical diagnosis* Overall 0.92 (0.73–1.15) Women 0.90 (0.69–1.17) Men 1.21 (0.76–1.93).	1988/1989–1998/1999 and 1999/2001– 2009/2010	10 years	Overall −3.5 % Women −3.8 %	Compared with the earlier cohort, the later cohort had more education, a higher BMI, a lower prevalence of stroke, and were less likely to be a current and more likely to be former smokers. More use of antihypertensive and lipid-lowering drugs. At baseline, they were less disabled on the 4-item IADL score and had higher MMSE scores. Differences in education, vascular factors and depression accounted only to some extent for this reduction (overall AHR 0.77, 95 % CI 0.61–0.97; women AHR 0.73, 95 % CI 0.57–0.95).
4. Rotterdam, the Netherlands, 60–90 years [39]	Dementia (DSM-III-R)	Non-significant 25 % reduction RR 0.75 (0.56–1.02)	1990–2000	10 years	−2.5 %	Hypertension, diabetes and obesity increased. Higher education. More diabetes treatment, more anti-thrombotics and much more statins. More past but less current smoking. Substantial reduction in overall mortality: HR 0.63 (0.52–0.77).
5. Germany, insurance claims data, 65 years and older [40]	Dementia (ICD-10), or using cholinesterase inhibitors or memantine	9 % reduction Men 0.91 (0.85–0.97) Women 0.91 (0.87–0.95)	2004–2007/2007–2010	3 years	−3.0 %	This study used claims data of the largest public health insurance company in Germany. The data contained complete inpatient and outpatient diagnoses according to ICD-10 codes. For the analysis of incidence, two independent age-stratified samples were taken, the first comprising 139,617 persons in 2004 with follow-up until 2007, the second with 134,653 persons in 2007 with follow-up until 2010. Secular trends in clinical diagnosis or help-seeking cannot be excluded.
6. Ontario, Canada; health insurance plan, hospital discharge and ambulatory care register; age range not reported [41]	Dementia diagnosis (ICD-9 or ICD-10) or cholinesterase inhibitor prescription	7.4 % reduction; statistical significance of trend not reported	2002–2013	12 years	−0.6 %	This study used claims data of the single state-provided insurance plan and comprehensive hospital admission, ambulatory care and drug prescription databases. Annual incidence rates, age- and sex-standardised, are reported for each year between 2002 and 2013. The trend is not linear, and statistical significance is not reported. Secular trends in clinical diagnosis or help-seeking cannot be excluded.
7. Chicago, IL, USA [31]	AD	Stable OR 0.97 (0.90–1.04)	1997–2008	11 years	No trend	
8. Ibadan, Nigeria [52]	Dementia (DSM-III-R) AD	Stable Dementia 1.7 % per annum (1.4–2.0 %) vs. 1.4 % per annum (1.1–1.6 %) AD 1.5 % per annum (1.2–1.8 %) vs. 1.0 % (0.7–1.2 %)	1991–2002	11 years	No trend	
Inferred						
9. Stockholm, Sweden, 75 years and older [29]	Dementia (DSM-III-R)	Reduced incidence inferred from stable prevalence but increased survival with dementia	1988–2002	14 years	Not reported	See also notes for Table 1, study 5.

*AD* Alzheimer’s disease, *AHR* adjusted hazard ratio, *BMI* body mass index, DSM *Diagnostic and Statistical Manual of Mental Disorders*, *HDL* high-density lipoprotein, *IADL* instrumental activities of daily living, *ICD* International Classification of Diseases, *MMSE* Mini Mental State Examination, *NINCDS-ADRDA* National Institute of Neurological and Communicative Disorders and Stroke and the Alzheimer’s Disease and Related Disorders Association, *NINDS-AIREN* National Institute of Neurological Disorders and Stroke and Association Internationale pour la Recherche et l’Enseignement en Neurosciences, *RR* relative risk, *VaD* vascular dementia

### Dementia mortality

In only four of the studies did the researchers take the opportunity to study or report changes in mortality and/or survival among people with dementia, or the ratio of mortality rates between those with and without dementia (Table 3). In the Rotterdam study [39], overall mortality had declined by 37 % in the 10 years between the two cohorts, but this was not reported with stratification by dementia status. In the HRS in the United States, as well as in the Stockholm study [29], the mortality ratio for dementia remained relatively stable over time, suggesting that, under the assumption that mortality rates were falling among those without dementia, there would have been similar rates of decline for those living with dementia. This was clearly demonstrated in the Stockholm study, where an absolute decline in mortality rates of 30 % over 14 years was seen for those with and without dementia, for both sexes [29]. The relationship between trends in prevalence, incidence and mortality remain unclear, partly because in most of the eligible studies only some of these parameters were directly observed. Only in the German insurance claims data were changes in prevalence, incidence and mortality reported, but these are mutually inconsistent, perhaps because different samples and time periods were used for the prevalence [32] and incidence and/or mortality trend analyses [40]. In marked contrast to other studies, a precipitous increase in mortality rates among people with dementia, particularly women, was noted over a short time interval [40]. In Stockholm (where prevalence and mortality were observed) [29], and in Indianapolis, IN, USA (where prevalence and incidence were observed) [31, 36], findings are consistent with declining incidence but stable prevalence, accounted for by increasing duration of dementia (declining dementia mortality).

**Table 3:** Changes in mortality among people with dementia

Study, setting, age range	Outcomes	Change in mortality and/or mortality hazard ratio	Period	Interval (years)	Other findings/notes
Directly observed					
1. United States, HRS [21]	Mortality hazard ratio	Non-significant increase, from HR 2.53 to 3.11, *p* = 0.09	1993–2002	9 years	No report of absolute mortality rates, stratified or unstratified. However, given a presumed decline in overall mortality, it seems likely that mortality also declined among people with dementia, but to a slightly lesser extent
2. Stockholm, Sweden [29]	Mortality hazard ratio	Stable HR: 2.42 (2.03–2.87) vs. 2.47 (2.03–3.00)	1988–2002	14 years	Secular trend similar (30 % reduction in mortality) to that for those with no dementia, and for both sexes
	Mortality rate among people with dementia	29 % reduction in mortality (HR 0.71, 95 % CI 0.57–0.88) adjusted for age, sex, education and MMSE score			
3. Germany, insurance claims data, 65 years and older [32]	Mortality rate among people with dementia	20 % increase in mortality among women with dementia (*p* < 0.0001) Non-significant 11 % increase in mortality among men with dementia (*p* = 0.75)	2004–2007	3 years	Mortality among women without dementia remained constant over the two cohorts, while there was a non-significant 4 % decline in mortality rates among men without dementia. These findings would suggest an increase in the mortality hazard ratio associated with dementia, greater for women than for men.
Inferred					
4. Indianapolis, IN, USA, African Americans, 65 years and older [31, 36]	Dementia duration	Increase in survival with dementia can be inferred from stable prevalence of dementia [31], but 55 % fall in incidence [36]	1991–2002	11 years	Extrapolation from reported prevalence and incidence rates at the two time points suggests that survival time with dementia is 2.4 times longer for the second cohort.

*MMSE* Mini Mental State Examination

### Secular trends within regions estimated from meta-analyses of individual studies

Another approach to estimating secular trends involves combining evidence from all studies conducted within a particular country or region, using a meta-analytical approach and meta-regression to estimate the effect of time of study upon prevalence. This approach was used in the 2009 ADI World Alzheimer Report to estimate secular trends in dementia prevalence in Europe [1]. One problem with such exercises is that, in contrast to the studies previously reviewed, which hold such factors constant, there is inevitably considerable heterogeneity in the nature of the population studied and the methods used for the surveys, which may in turn affect the prevalence recorded. It is therefore important, to the extent possible, to control for such effects in the meta-regression. In the European meta-analyses, there was no evidence for a trend in prevalence between 1980 and 2008 [4], and this held true when the evidence base was updated to include studies conducted through 2015 for the 2015 World Alzheimer Report [3].

East Asia is the one other world region with sufficiently numerous prevalence studies to permit meta-regression and estimation of secular trends in dementia prevalence. A study of secular trends in Japan (part of the adjacent Asia Pacific high-income region) reported a tendency towards increasing prevalence, but this was based on only eight data points, including the four waves of the Hisayama study [34], and did not control for study methodology [43]. A systematic review and meta-analysis of 11 population-based prevalence studies conducted in South Korea since 1990 identified a trend towards a decrease in the prevalence of dementia until 2000–2005, with a subsequent increase, but with no statistically significant temporal variation, having adjusted for sample composition and study methodological quality [44]. The East Asia evidence base and the population of older people at risk is dominated by China, the focus of one recent meta-analysis [45], while a second meta-analysis also included studies conducted in Hong Kong and Taiwan [46]. Estimates taken from the China meta-analysis suggested a 46 % relative increase in age-standardised prevalence from 1990 to 2010 (+2.3 % per year), while in the wider review the increase was 171 % from studies conducted in the pre-1990 period to 2005–2012 (a prevalence of 2.1 % pre-1990, 3.4 % for 1990–1994, 3.9 % for 1995–1999, 4.4 % for 2000–2004 and 5.7 % for 2005–2012). However, in that study, the secular trend was considerably reduced, to 72 %, and was no longer statistically significant, having controlled for study methodology (1.8 % pre-1990, 2.5 % for 1990–1994, 2.1 % for 1995–1999, 2.4 % for 2000–2004 and 3.1 % for 2005–2012).

The most important potential confounder appeared to be the choice of dementia diagnostic criteria. Older studies tended to use criteria from the *Diagnostic and Statistical Manual of Mental Disorders, Third Edition* (DSM-III), the DSM-III-R, or the International Classification of Diseases, Tenth Revision, which then tended to record a lower prevalence of dementia than those more recent studies that used DSM-IV dementia, 10/66 dementia criteria or Geriatric Mental State Examination (GMS)-Automated Geriatric Examination for Computer Assisted Taxonomy (AGECAT) criteria. For the purposes of estimating current dementia prevalence, whether the higher estimates for the most recent period are explained by real underlying secular trends or the use of more up-to-date and valid diagnostic criteria, or both, is immaterial. However, for the purposes of forecasting future trends in prevalence and numbers in the region, the distinction is clearly crucially important [47]. As previously indicated, there is evidence that cardiovascular health is deteriorating among older people in China [11], a trend also evident in other middle-income countries [9]. The prevalence of smoking among adult men in China is among the highest in the world, and an epidemic among younger women is well underway [48]. Rapid dietary transition is leading to an epidemic of obesity and cardiometabolic disease [49]. A recent modelling exercise assessed the likely impact of recent increases in obesity among middle-aged Chinese on dementia prevalence, assuming a causal link with dementia. The authors of that study concluded that future dementia prevalence in China may have been underestimated by up to 19 %, given the additional impact of epidemiologic transition [50]. The relative contributions of changes in diagnostic criteria, as well as changes in risk factor exposure, both associated with the time that the study was conducted, are uncertain and cannot be resolved with currently available data.

## Discussion

We have updated our recent work on the global burden of dementia and reviewed the entirety of the current global evidence on trends in the prevalence, incidence, and survival with or mortality due to dementia, using data from studies in which investigators had monitored these indicators over time in defined populations with fixed survey and dementia ascertainment methodologies. We also reviewed regional meta-analyses in which researchers had sought to estimate regional trends in prevalence across studies, conducted in various sites and using diverse methods. The present review is the most comprehensive such study to date.

There is no clear evidence from this review to justify a departure from the current position of assuming constant age-specific dementia prevalence when making projections of the numbers likely to be affected in the future [3]. The evidence for a declining trend in the incidence of dementia, at least in high-income countries, is somewhat more consistent, although still patchy, and as yet thinly evidenced. Although the evidence on changes in survival in those with dementia is extremely limited, it is plausible that the effects of a reduced incidence upon prevalence are likely to be offset by a longer survival of those living with dementia.

### Potential for prevention

The future course of the global dementia epidemic through to 2050 is likely to depend, at least to some extent, upon the success or otherwise of continuing efforts to improve public health [7, 25]. Those who will be old in 2050 were born around the 1970s and have already received their basic education. They are now in their fourth and fifth decades of life, a crucial ‘sensitive period’ in which evidence suggests that efforts to prevent, detect and control obesity, hypertension, diabetes and dyslipidaemia are likely to have maximum positive impact upon brain health and dementia risk in later life [7, 25]. Such public health strategies, alongside secular improvements in education, are plausibly likely to result in a progressive decline in age-specific incidence of dementia in high-income countries, the magnitude of which is currently uncertain.

### The important impact of survival with dementia

Whether declining incidence is accompanied by a decline in the age-specific prevalence of dementia will depend upon any coincident changes in survival and/or mortality patterns of people living with dementia, which are difficult to predict on the basis of current data. If the onset of dementia occurs close to the end of the natural lifespan, fewer years may be lived with dementia. Two studies suggest that decline in incidence may be greater in younger age groups, suggesting that the incidence of dementia may be being deferred into older age [51, 52]. This may be consistent with the observation of an increasing prevalence of dementia among the oldest old in one Swedish study [33], but it is inconsistent with the observation from the MRC CFAS study of greater reductions of dementia prevalence among older age groups [27]. Since most of the public health interventions that have been proposed to reduce the incidence of dementia (for example, tobacco control, and prevention and treatment of hypertension) also have benefits in reducing incidence and mortality due to other chronic diseases, one should expect that reductions in prevalence arising from reduced incidence of dementia may be offset, at least to some extent, by reduced mortality and longer survival with dementia [53]. Most of the more plausible scenarios are more consistent with either a stable or a modestly increasing disease prevalence [53, 54]. Of concern, current evidence of adverse trends in cardiovascular risk factors and morbidity in low- and middle-income countries are consistent with a future increase in age-specific incidence and prevalence of dementia in those regions.

Other factors, such as improvements in standards of health and social care for people with dementia as well as provision or withholding of life-prolonging critical interventions, might also be expected to have an influence on mortality rates among people living with dementia. In well-resourced, advanced healthcare settings, there is growing awareness that critical interventions should not be withheld simply because someone has dementia when these would improve quality of life. At the same time, in the context of end-of-life care, the focus should be on palliation to improve quality of life, and interventions that merely prolong life with risk of harm to the patient should be withheld [55]. In low- and middle-income countries, there is evidence that people with dementia currently have particular problems in accessing healthcare that might benefit their health and survival [56].

### Implications for future research

Studies that use fixed methodology to estimate changes in dementia prevalence, incidence and mortality over time, in defined populations, are uniquely valuable assets. It is important that more such studies be commissioned. The most valuable will be those that track all three parameters over time, which none of the studies reviewed in this paper did. Surveys with nationally representative samples will have the greatest generalisability and the greatest potential to both inform and track the impact of national policies. Where trends are observed, it will be important to relate these to compositional changes in the population, particularly to changes in levels of exposure to critical risk factors. However, in very few studies have researchers made a comprehensive assessment of such compositional factors and their changes over time, and in only three studies did investigators attempt to attribute changes in dementia frequency to changes in risk factor exposure [21, 37, 38]. It is clearly important that such studies do, as far as possible, hold methodology constant. Several of those reviewed here did make small changes between waves, the effect of which upon the observed trends cannot be determined with complete confidence [27, 31]. Diagnostic criteria change over time, but these too must be held constant to make meaningful comparisons, a problem that can be surmounted by using the updated criteria alongside the original criteria, where feasible and appropriate. A more intractable problem is the probable changes in clinician training, practice and opinions regarding the operationalisation of diagnostic criteria [38, 47]. This may also be countered through the application of structured assessments and diagnostic algorithms, such as the AGECAT computerised algorithm linked to the Geriatric Mental State [57], as employed in the MRC CFAS studies [27], or the 10/66 Dementia Research Group’s cross-culturally validated diagnostic algorithm [58, 59]. Finally, the potential for selection bias due to declining participation rates and increasing attrition rates in cohort studies needs to be carefully considered [27].

In previous modelling exercises, researchers have sought to predict what might happen to the future prevalence of dementia, given our best estimates of risk associations and possible changes in those risk factor profiles over time [8, 50]. In the light of the present review, these estimations appear overoptimistic. An alternative approach is to observe and correlate actual changes in risk factor profiles and dementia incidence over time. This is a well-established modelling approach in the cardiovascular disease field and has contributed greatly to understanding of the potential for prevention, and the attribution of changes in disease incidence to specific factors, to further guide prevention strategies [60–62]. Of note, the three studies in this review in which investigators attempted to do this indicate that changes in education and cardiovascular risk account for only a modest proportion of observed reduction in prevalence or incidence [21, 37, 38]. The interesting implication would be that other unanticipated, unmeasured and uncontrolled secular changes in population characteristics may have had an important impact. Similar studies should be carried out in the future to monitor the impact of prevention programs on the future scale of the dementia epidemic.

## Conclusions

The best available evidence suggests that the age-specific prevalence of dementia is unlikely to change significantly in coming years, even if the incidence of dementia falls in response to secular improvements in public health in high-income countries. This conclusion remains provisional, given the limited data available on secular trends and the heterogeneity in study findings. Prudent policymakers should exercise due caution, being swayed neither by individual studies nor by Pollyannaish statements, such as expressed in a recent *Lancet* editorial [63]:

The projections of the ADI report for 2050 are alarming, but it is important to bear in mind that they are just that – projections.... The opportunity is here to ensure that the grim outlook for dementia in 2050, especially in low-income and middle-income countries, becomes nothing more than a work of fiction.

Future projections [3] may actually turn out to be conservative, particularly for low- and middle-income countries, should effective public health action not be taken. Under currently foreseeable scenarios, they should be considered as constituting the mid range of expectations. More research into national and regional trends in disease frequency, linked to changes in exposure levels to known risk factors, is urgently required.

## Abbreviations

AD: Alzheimer’s disease
ADI: Alzheimer’s Disease International
AGECAT: Automated Geriatric Examination for Computer Assisted Taxonomy
AHR: adjusted hazard ratio
AOR: adjusted odds ratio
BMI: body mass index
DSM: *Diagnostic and Statistical Manual of Mental Disorders*
GMS: Geriatric Mental State Examination
HDL: high-density lipoprotein
HRS: Health and Retirement Study
IADL: instrumental activities of daily living
ICD: International Classification of Diseases
MMSE: Mini Mental State Examination
MRC CFAS: Medical Research Council Cognitive function and Ageing Study
NINCDS-ADRDA: National Institute of Neurological and Communicative Disorders and Stroke and the Alzheimer’s Disease and Related Disorders Association
NINDS/AIREN: National Institute of Neurological Disorders and Stroke and Association Internationale pour la Recherche et l’Enseignement en Neurosciences
RR: relative risk
VaD: vascular dementia
